# Effects of whey protein supplementation prior to, and following, resistance exercise on body composition and training responses: A randomized double-blind placebo-controlled study

**DOI:** 10.20463/jenb.2019.0015

**Published:** 2019-06-30

**Authors:** Yeram Park, Hun-Young Park, Jisu Kim, Hyejung Hwang, Yanghoon Jung, Richard Kreider, Kiwon Lim

**Affiliations:** 1 Department of Physical Education, Konkuk University, Seoul Republic of Korea; 2 Physical Activity and Performance Institute, Konkuk University, Seoul Republic of Korea; 3 CJ Research Institute, CJ CheilJedang, Suwon Republic of Korea; 4 Department of Health and Kinesiology, Texas A&M University, Texas U.S.A.

**Keywords:** Protein supplementation, training, muscular strength, muscular endurance, hormonal response

## Abstract

**[Purpose]:**

The composition of protein supplements, the consumption timing immedi¬ately before and after resistance exercise training (RET), and the quantity of protein supplementation may be important factors for the im-provement of muscle mass and function. Although these factors should be considered comprehensively for effective improvement of muscular function in protein supplementation, relatively few studies have focused on this area. Therefore, this study was designed to investigate whether a protein blend supplement before and after resistance exercise for 12 weeks would be effective in increasing muscular function.

**[Methods]:**

In total, 18 participants were randomly assigned to a placebo (PLA) or protein blend supplement (PRO) group. All subjects followed the same training routine 3 times per week for 12 weeks, taking placebo or protein supplements immediately before and after each exercise session. The protein supplement consisted of 40 g of blend protein, including hydrolyzed whey protein. The RET consisted of lower body (barbell squat, dead lift, seated leg extension, and lying leg curl) and upper body (bench press, barbell rowing, preacher bench biceps curl, and dumbbell shoulder press) exercises. A repetition was defined as three sets of 10–12 times with 80% of one repetition maximum (1RM).

**[Results]:**

Although the PRO group had a lower protein intake in terms of total food intake than the PLA group, the mean changes in muscle circumference, strength, and exercise volume increased, especially at week 12, compared to the PLA group.

**[Conclusion]:**

These results suggest that the composition and timing of protein intake are more important than the total amount.

## INTRODUCTION

The net protein balance (NPB) is defined as muscle protein synthesis (MPS) minus muscle protein breakdown (MPB). Thus, a significant rise in skeletal MPS (anabolism) and/or reduction in MPB (catabolism) can result in an increase in skeletal muscle mass. It has been clearly demonstrated that resistance exercise training (RET) stimulates a significant increase in MPS when the protein intake is sufficient. Thus, RET is generally considered to be a reliable strategy for improving muscle mass and function^1^.

Protein intake is important to maximize muscular function during resistance exercise. Based on the reports of the food and Nutrition Board of the Institute of Medicine, the dietary reference intake (DRI) is 56 g per day for men aged 19–29 years (Americans and Canadians). However, this is not sufficient to have a positive effect on the intracellular essential amino acid supply, prevent protein oxidation, recover muscle damage during exercise, and increase muscle mass and strength^2^. Therefore, protein supplementation is required to maximize the adaptive response of the skeletal muscle to prolonged RET. On the other hand, the net muscle protein balance remains negative in the absence of nutrient intake, indicating that the muscle tissue remains in a catabolic state^3^.

Recently, the general population, as well as athletes, are performing RET combined with protein supplementation in order to increase muscle mass and hypertrophy. Whey protein hydrolysate (WPH) was the most common type of protein supplements, followed by creatine, amino acid mixtures, and BCAA^4^. Protein supplementation induces protein synthesis by increasing components of the anabolic hormone response, such as growth hormone (GH), insulin-like growth factor (IGF-1), and testosterone (testo)^5^. Especially, leucine, which is abundant in WPH and one of BCAA, stimulates muscle protein synthesis, which subsequently improves muscle hypertrophy and function^6^. 

Moreover, the timing, amount, and type of dietary protein supplements are very important to maximize the effect of dietary protein supplements. In previous studies, the intake of whey and amino acids before resistance exercise stimulates a positive net protein balance^7^. It has also been demonstrated that an intake of whey and leucine immediately after resistance exercise leads to an anabolic hormonal response^8^. Dietary protein supplementation before and after resistance exercise increases post-exercise protein synthesis as well as net muscle protein during recovery^9^. In a study of the adequate protein intake, the changes in muscle protein synthesis according to the amount of protein intake (0, 5, 10, 20, and 40 g) after resistance exercise showed a plateau phenomenon from 20 g, indicating that 20 g of protein is required in order to stimulate muscle protein synthesis^10^. Moreover, the protein composition is important because the function of protein varies depending on its component. Protein can be hydrolyzed during manufacturing, producing smaller peptides that may accelerate the absorption and utilization of amino acids. Thus, it is possible that WPH may elicit an enhanced effect on muscle hypertrophy when combined with chronic RET^11^. In addition, the combined ingestion of whey proteins, BCAA, or creatine is more effective in increasing post-exercise muscle protein synthesis than protein alone^12^. In a study of the BCAA intake required to maximize muscle function, it was demonstrated that at least 5 g (0.077 to 0.087 g/kg) of BCAA before and after each exercise session was necessary^13^. Also, males who supplemented with a combination of whey protein and creatine had a greater increase in fat free mass and relative the 1RM of bench press than whey protein alone^14^. These studies suggest that the composition of the protein supplement, the consumption timing immedi¬ately before and after RET, and the quantity of the protein supplement may be important factors for the im¬provement of muscle mass and muscular function. However, there have been relatively few studies that have fully examined all of these factors. Therefore, the purpose of this study was to assess whether a protein blend supplement before and after resistance exercise for 12 weeks would be effective in terms of increasing muscle function.

## METHODS

### Experimental procedures

Each subject participated in four trials (0, 4, 8, and 12 weeks). The subjects were instructed to refrain from heavy physical activity for 48 h before testing, and to keep their diets as constant as possible. Participants presented to the laboratory after an 8 h fast and donated 5 ml of blood via venipuncture. Following blood collection, the subjects’ resting heart rate (HR), blood pressure (BP), body composition, muscle circumference, muscular strength and endurance were measured. The subjects completed these assessments and recorded a food log at 0, 4, 8, and 12 weeks of training.

### Participants

A total of 24 young men, with no history of participating in any regular exercise program, were included in the study. In order to be eligible for inclusion in the study, the participants had to be healthy and without any injuries to the musculoskeletal system that could interfere with the execution of training. All subjects were informed about the nature and risks of the experimental procedures before their informed consent was obtained. This study was approved by the Institutional Review Board of the Konkuk University (permit number: 7001355-2017-05-HR-176), and was registered with clinicaltrials.gov (#PRE20181025-004). Four participants in the placebo group (PLA) withdraw because of muscular in¬jury or moving to a new house, and two participants in the protein group (PRO) voluntarily withdrew because of personal reasons. Therefore, a total of 18 participants were included in the final analysis.

### Dietary supplementation

**Figure 1.**
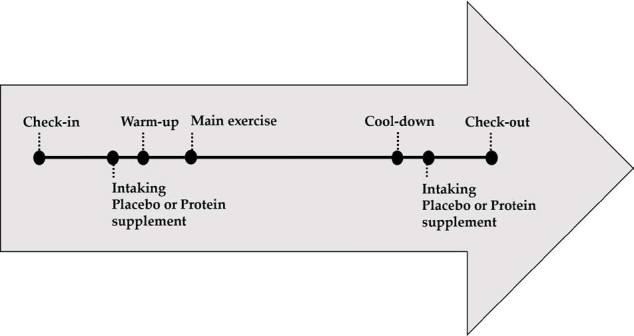
Overview of training day

Figure 1 presents an overview of the training day. On training days, supplementation was given immediately before and after each exercise session (3 sessions/week). The protein supplement consisted of 40 g of a proprietary blend protein (26.4 g of WPH and WPC, including 5.8 g of BCAA), 1 g creatine monohydrate, 8 g carbohydrate, and 2 g fat. The PLA group were given an identical amount of calories, including carbohydrates, and 2.7 g of other components (cocoa powder, vegetable cream, xanthan gum, and saccharide). The other components were identical in nature and quantity in the two products. The supplements were stored in identical opaque sachets in order to render identification of the respective supplements difficult. In order to maintain the double-blind nature of the study, neither the subjects nor any of the involved researchers knew which group the subjects belonged to. The subjects were allowed to drink water before, during, and after each exercise session.

### Exercise program

The 1RM tests were repeated every 4 weeks (0, 4, 8, and 12 weeks), and 1RM was indirectly determined through the repetition of the submaximal load because participants with no experience of any other exercise are likely to be injured. The 1RM was predicted by substituting the weight and the maximum number of repetitions that could be repeated approximately 8–10 times into the 1RM estimation formula. The estimation equation 1RM = weight lifted (kg)/[1.0278 - (number of repetitions)] used in this study was developed by Brzycki^15^. In the training program, the intensity of exercise was 80% of 1RM, and the intensity of 1RM was progressively increased by 20% in the upper and lower body every 4 weeks throughout the training program.

All participants were required to follow the same resistance training routine, which consisted of 3 days per week split into four lower body (barbell squat, dead lift, seated leg extension, and lying leg curl) and four upper body (bench press, barbell rowing, preacher bench biceps curl, and dumbbell shoulder press) exercises, for a total of 12 weeks. Each exercise consisted of three sets of 10–12 repetitions performed with 80% of 1RM. The total exercise duration was approximately 60 min, and sessions were supervised by a trainer who provided written verification that the work out was completed. The resistance training program is shown in Table 1. 

**Table 1.** Resistance training program

Type	Content		Intensity
Warm-up (10 min)	Upper body stretching	Back, chest, biceps brachii, triceps brachii, shoulder	20 sec x 3 sets
	Lower body stretching	Quadriceps, hamstring, hip, hip joint	
Main exercise (40–50 min)	Upper body resistance exercise	Bench press, biceps curl, barbell row, shoulder press	1RM 80% 10–12 rep. x 3 sets Rest: 1 min
	Lower body resistance exercise	Leg extension, leg curl dead lift, barbell squat	
Cool-down (10 min)	Upper body stretching	Back, chest, biceps brachii, triceps brachii, shoulder	20 sec x 3 sets
	Lower body stretching	Quadriceps, hamstring, hip, hip joint	

Min: Minute, Sec: Second, Rep: Repetition

### Diet assessment

Prior to trial, subjects recorded a food log for 3 days (i.e. 2 days before, and on the trial day); this served to standardize the food intake throughout the intervention period, which was performed every 4 weeks. Participants were requested to maintain their diet habits throughout the experimental period, and were educated on the method of diet recording (the amount of all foods consumed, including both meals and snacks). A trained dietician analyzed the nutrient content using software CAN-pro 5.0 (Computer Aided Nutritional analysis program, version 5; The Korea Nutrition Society, 2015). From this analyzer, the daily food intake in terms of total energy, carbohydrate, protein, and fat was determined.

### Body composition

The subjects’ body composition was measured every 4 weeks by bioimpedance. The X-Scan plus II body composition analyzer (Jawon Medical Company, Republic of Korea) was used to determine the subjects’ height, weight, fat free mass (FFM), and fat mass (FM), with an accuracy of SD ± 1% based on body fat (%). The subjects were instructed to stand on the foot platforms of the instrument and hold the palm-and-thumb electrodes with their arms not touching their torso. 

### Muscle circumference

In order to assess changes in the hypertrophy of the upper arm and lower thigh following 12 weeks of resistance training, the muscle circumference was measured with a tape every 4 weeks; the use of tape is a convenient tool for measuring circumference due to its pull-out mechanism that can be used for precise measurement around the body. After sufficient stabilization, the mean value of two measurements was used. The length of the upper arm (right-mid-arm and left-mid-arm) and the lower thigh (right-mid-thigh and left-mid-thigh) was measured. Then, the circumference of the midpoint was measured^16^.

### Muscular strength

Maximum strength was assessed by one-repetition maximum (1RM) for the upper body (bench press, bent over barbell row, preacher bench biceps curl, and dumbbell shoulder press) and lower body (barbell squat, barbell deadlift, leg extension, and leg curl). Before the 1RM was measured, the subjects performed 10 repetitions of warm-up exercises at 50% of the perceived 1RM and stretching of the major muscle groups. Three maximal trials, each separated by 3 min of rest, were adjusted in order to determine the individual 1RM for each resistance exercise^17^.

### Muscular endurance

The muscular endurance was evaluated by the maximal number of repetitions at 70% of 1RM in the upper body (bench press, bent over barbell row, preacher bench biceps curl, and dumbbell shoulder press) and lower body (barbell squat, barbell deadlift, leg extension, and leg curl) until exhaustion. Muscular exhaustion was defined as the moment when the weight ceased to move, or the subjects failed to maintain the prescribed pace. Any repetition that lacked the full range of movement was not counted. Following the test, the performed exercise volume (EV) was calculated (load x repetition) and used as a measure of muscular endurance^18^.

### Blood analyses

The hormonal response was analyzed by the Green Cross Medical Foundation (an organization certified by The Korea Society for Laboratory Medicine). The concentration of the following blood variables was quantified: GH, IGF-1, vascular endothelial growth factor (VEGF), testo, and cortisol. A 5-mL sample of venous blood was collected into serum separating tubes (SST) and heparin tubes. Blood serum was obtained by clotting for 30 min at room temperature before centrifugation. Both serum and plasma were centrifuged at 4 °C for 15 min at 3000 rpm and stored at -80 °C until analysis.

Plasma samples were analyzed for the concentration of GH and IGF-1 using a chemiluminescent immunoassay (CLIA) with Immulite 2000 GH (Siemens Healthcare Diagnostics, Malvern, USA) and LIAISON IGF-1 (Diasorin, Saluggia, Italy), respectively. The blood VEGF level was measured by Enzyme-Linked Immunosorbent Assay (ELISA) with a human VEGF Quantikine ELISA Kit (R&D system, Minneapolis, USA). Plasma testosterone and cortisol concentrations were analyzed using a Chemiluminscent Microparticle Immunoassay (CMIA) with Architect® 2nd Generation Testosterone and Architect® Cortisol (Architect, Abbott Diagnostics, Abbott Park, IL, USA). The test-to-test reliability assessment of the assays evaluated in the study yielded the following mean CVs: GH: 4.55–6.56, IGF: 7.03–8.66, VEGF: n/a, testo: 5.51–8.18, and cortisol: 3.41–7.3).

### Statistical analyses 

The baseline demographic was analyzed by one-way analysis of variance (ANOVA). All data were analyzed using general linear models (GLM) and multivariate analysis of variance (MANOVA) with repeated measures, with Wilks’ Lambda and Greenhouse-Geisser adjustments. The data were also graphed with means and 95% confidence intervals (CI) in order to determine whether the changes from baseline were significant. All data are presented as mean ± SD, or mean change and 95% CI.

## RESULTS

### Baseline characteristics

Table 2 presents the participant demographics by group assignment. A total of 18 participants completed the study (PLA = 8, PRO = 10). One-way ANOVA analysis revealed no significant differences between groups with regards to baseline age, height, body weight, body mass index (BMI), FFM, and FM.

**Table 2.** Participant characteristics

	PLA (n = 8)	PRO (n = 10)	*p*-value
Age (years)	23.3 ± 3.1	24.6 ± 1.8	0.262
Height (cm)	174.6 ± 8.0	175.4 ± 7.8	0.827
Body weight (kg)	76.7 ± 10.3	70.8 ± 10.7	0.254
BMI (kg/m2)	25.1 ± 2.6	22.6 ± 2.9	0.071
Fat free mass (kg)	58.9 ± 6.7	58.1 ± 6.3	0.790
Fat mass (kg)	17.8 ± 4.8	12.7 ± 6.5	0.085

Min: Minute, Sec: Second, Rep: Repetition

### Dietary intake

Table 3 presents the 3 day diet assessment data observed between groups at 0, 4, 8, and 12 weeks of training. MANOVA analysis revealed no significant group x time interactions between groups in terms of relative energy intake (p = 0.063), protein intake (p = 0.250), carbohydrate intake (p = 0.087), and fat intake (p = 0.544). However, the daily quantity of protein intake increased steadily in the PLA group but not the PRO group. Moreover, energy intake including the quantity of protein supplement was not increased in the PRO group (week 0: 26.02 ± 7.25; week 4: 25.93 ± 8.25; week 8: 24.51 ± 8.74; and week 12: 25.35 ± 6.82), and it was less than PLA group (data not shown).

**Table 3.** Diet assessment

Variable	Group	Time (wk)					*p*-value
		0	4	8	12		
Energy intake (kcal/kg/d)	PLA	23.08 ± 9.75	28.18 ± 8.02	34.04 ± 9.45 ^a^	28.47 ± 5.84	Time	0.223
						Group	0.307
	PRO	25.67 ± 7.56	25.56 ± 8.51	24.12 ± 8.91	24.94 ± 6.93	G x T	0.063
Carbohydrate (g/d/kg)	PLA	2.84 ± 0.87	3.22 ± 0.87	3.44 ± 0.88	3.72 ± 0.57 ^a^	Time	0.293
						Group	0.637
	PRO	3.20 ± 0.85	3.22 ± 1.38	2.92 ± 0.92	3.11 ± 0.77	G x T	0.087
Protein (g/d/kg)	PLA	0.76 ± 0.36	1.05 ± 0.48	1.26 ± 0.39 ^a^	0.88 ± 0.15 ^c^	Time	0.090
						Group	0.813
	PRO	0.93 ± 0.21	1.04 ± 0.42	0.97 ± 0.39	0.90 ± 0.20	G x T	0.250
Fat (g/d/kg)	PLA	0.87 ± 0.47	1.17 ± 0.51	1.19 ± 0.39	0.91 ± 0.22	Time	0.334
						Group	0.348
	PRO	0.91 ± 0.30	0.91 ± 0.36	0.95 ± 0.48	0.88 ± 0.28	G x T	0.544

Values are presented as means ± standard deviations. All variables were analyzed by MANOVA. MANOVA analysis revealed overall Wilks’ Lambda group (p = 0.39), time (p = 0.10), and group x time (p = 0.06). Greenhouse-Geisser time and group x time interaction p-values are reported with univariate group p-values. PLA: Placebo group, PRO: Protein supplement group.

^a^ represents p < 0.05 difference from baseline

^b^ represents p < 0.05 difference from wk 4

^c^ represents p < 0.05 difference from wk 8

### Muscle circumference 

**Figure 2.**
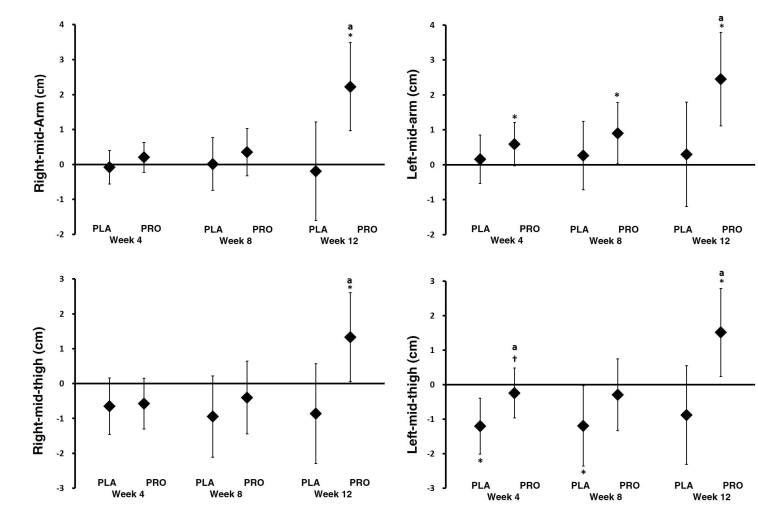
Muscle Circumference. Data are mean change and 95% CI. * represents p <0.05 difference from baseline. ^a ^represents *p* <0.05 from PLA. † represents *p*>0.05 to *p*<0.1

MANOVA analysis revealed significant time effects in terms of changes in right-mid-arm (1.01 ± 0.44 kg, p = 0.037), left-mid-arm (1.37 ± 0.47 kg, p = 0.010), and left-mid-thigh (0.31 ± 0.45 kg, p = 0.502) muscle circumference (Table 4). In addition, univariate interactions were observed between groups in all variables. As can be seen in Figure 2, the mean changes in muscle circumference were generally increased, with 95% CI’s above baseline, in the PRO group compared to the PLA group which had lower values, with 95% CI’s crossing baseline. More specifically, comparisons at week 12 demonstrated a significant increase in right-mid-arm circumference for the PRO group (2.22 cm, 95% CI, 0.96–3.48), but not for the PLA group (-0.19 cm, 95% CI, -1.60–1.21). For the left-mid-arm circumference assessment, the 4, 8, and 12 week changes were as follows: PRO group (week 4: 0.59 cm, 95% CI, -0.02–1.20; week 8: 0.90 cm, 95% CI, 0.02–1.77; week 12: 2.45 cm, 95% CI, 1.11–3.78), and PLA group (week 4: 0.15 cm, 95% CI, -0.53–0.84; week 8: 0.26 cm, 95% CI, -0.71–1.24; and week 12: 0.29 cm, 95% CI, -1.19–1.78). For the right-mid-thigh circumference assessment, the week 12 changes were significant for the PRO group (1.33 cm, 95% CI, 0.05–2.60), but not the PLA group (-0.86 cm, 95% CI, −2.29–0.56). For the left-mid-thigh circumference assessment, the week 12 changes were significant for the PRO group (1.51 cm, 95% CI, 0.21–2.80), but not for the PLA group (-0.88 cm, 95% CI, -2.32–0.56). In addition, there were between-group differences in all variables at 12 weeks, and there tended to be a difference in the left-mid-thigh measurement at 4 weeks.

**Table 4.** Muscle circumference

Variable	Group	Time (wk)					*p*-value
		0	4	8	12		
Right mid arm (cm)	PLA	32.1 ± 2.2	32.1 ± 2.0	32.2 ± 2.1	32.0 ± 1.5	Time	0.028^†^
						Group	0.41
	PRO	30.5 ± 2.9	30.7 ± 2.4	30.9 ± 2.1	32.8 ± 2.1 ^abc^	G x T	0.010^†^
Left mid arm (cm)	PLA	31.9 ± 2.1	32.0 ± 1.9	32.1 ± 1.6	32.2 ± 1.2	Time	0.008^†^
						Group	0.25
	PRO	29.8 ± 3.0	30.4 ± 2.4	30.7 ± 2.3 ^a^	32.3 ± 2.1 ^abc^	G x T	0.033^†^
Right mid thigh (cm)	PLA	52.7 ± 3.2	52.1 ± 3.6	51.8 ± 3.2	51.9 ± 1.8	Time	0.074
						Group	0.65
	PRO	51.3 ± 4.0	50.7 ± 3.9	50.9 ± 3.2	52.6 ± 3.0 ^abc^	G x T	0.037^†^
Left mid thigh (cm)	PLA	52.6 ± 3.4	51.4 ± 3.3 ^a^	51.4 ± 2.9 ^a^	51.7 ± 2.8	Time	0.023^†^
						Group	0.75
	PRO	51.1 ± 3.8	50.8 ± 3.3	50.8 ± 2.7	52.6 ± 3.0 ^abc^	G x T	0.036^†^

Values are presented as means ± standard deviations. All variables were analyzed by MANOVA. MANOVA analysis revealed overall Wilks’ Lambda group (p = 0.54), time (p < 0.001), and group x time (p = 0.06). Greenhouse-Geisser time and group x time interaction p-values are reported with univariate group p-values. * represents p < 0.05 from PLA.

^a^ represents p < 0.05 difference from baseline

^b^ represents p < 0.05 difference from wk 4

^c^ represents p < 0.05 difference from wk 8

^†^Significant interaction or main effect, p <0.05

### Muscle function 

**Figure 3.**
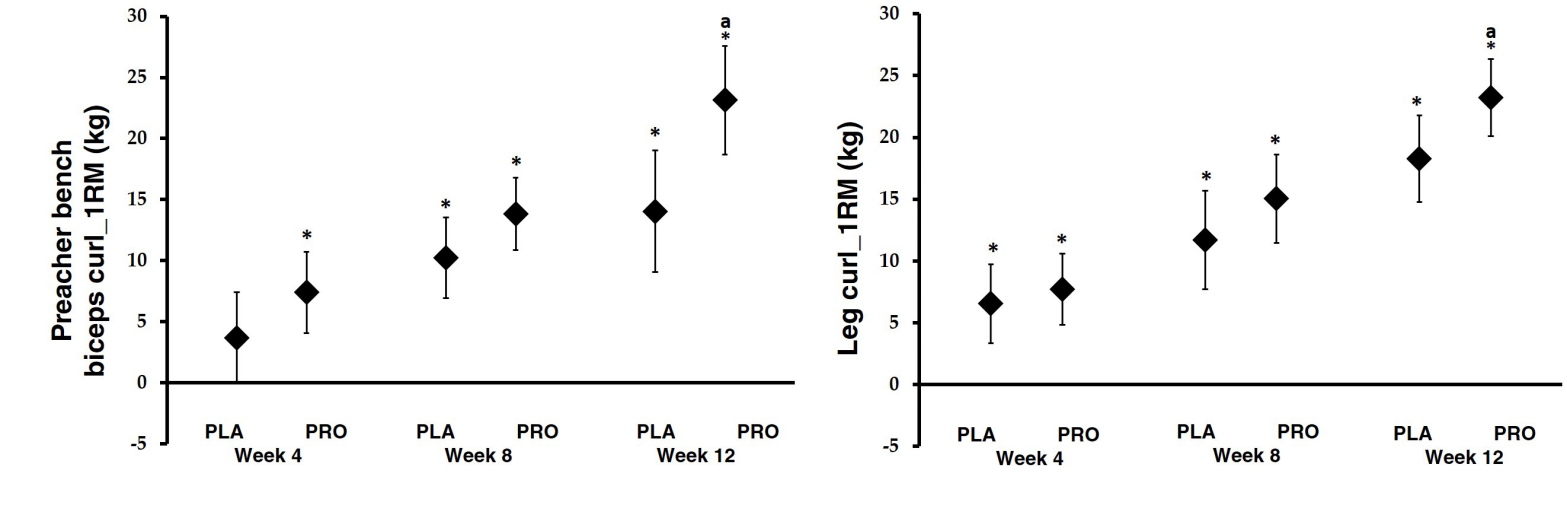
Muscular strength on Preacher bench biceps curl and Leg curl body. Data are mean change and 95% CI. * represents *p* <0.05 difference from baseline. ^a^ represents *p* <0.05 from PLA

With regards to upper and lower body muscle strength, MANOVA analysis revealed a significant time effect in changes in all variables. In addition, there were significant interactions observed between groups in the preacher bench biceps curl (p = 0.01) and dumbbell shoulder press (p = 0.01). As can be seen in Figure 3, for the preacher bench biceps curl 1RM assessment comparison, the 4-, 8-, and 12-week changes were as follows: PRO group (week 4: 7.39 kg, 95% CI, 4.05–10.73; week 8: 13.81 kg, 95% CI, 10.85–16.77; and week 12: 23.14 kg, 95% CI, 18.71–27.58), and PLA group (week 4: 3.68 kg, 95% CI, -0.05–7.42; week 8: 10.22 kg, 95% CI, 6.91–13.53; and week 12: 14.04 kg, 95% CI, 9.08, 19.00). There was also a difference in preacher bench biceps curl between groups at 12 weeks.

In terms of the lower body, as can be seen in Figure 3, the 4, 8, and 12 week changes in leg curl 1RM were as follows: PRO group (week 4: 7.70 kg, 95% CI, 4.83–10.57; week 8: 15.03 kg, 95% CI, 11.47–18.60; and week 12: 23.22 kg, 95% CI, 20.09–26.35) and PLA group (week 4: 6.53 kg, 95% CI, 3.32–9.74; week 8: 11.68 kg, 95% CI, 7.69–15.66; and week 12: 18.28 kg, 95% CI, 14.78–21.78). The barbell squat, barbell deadlift, and leg extension results showed a similar trend to the leg curl results. In addition, there was a difference in leg curl measurements between groups at 12 weeks. 

**Figure 4.**
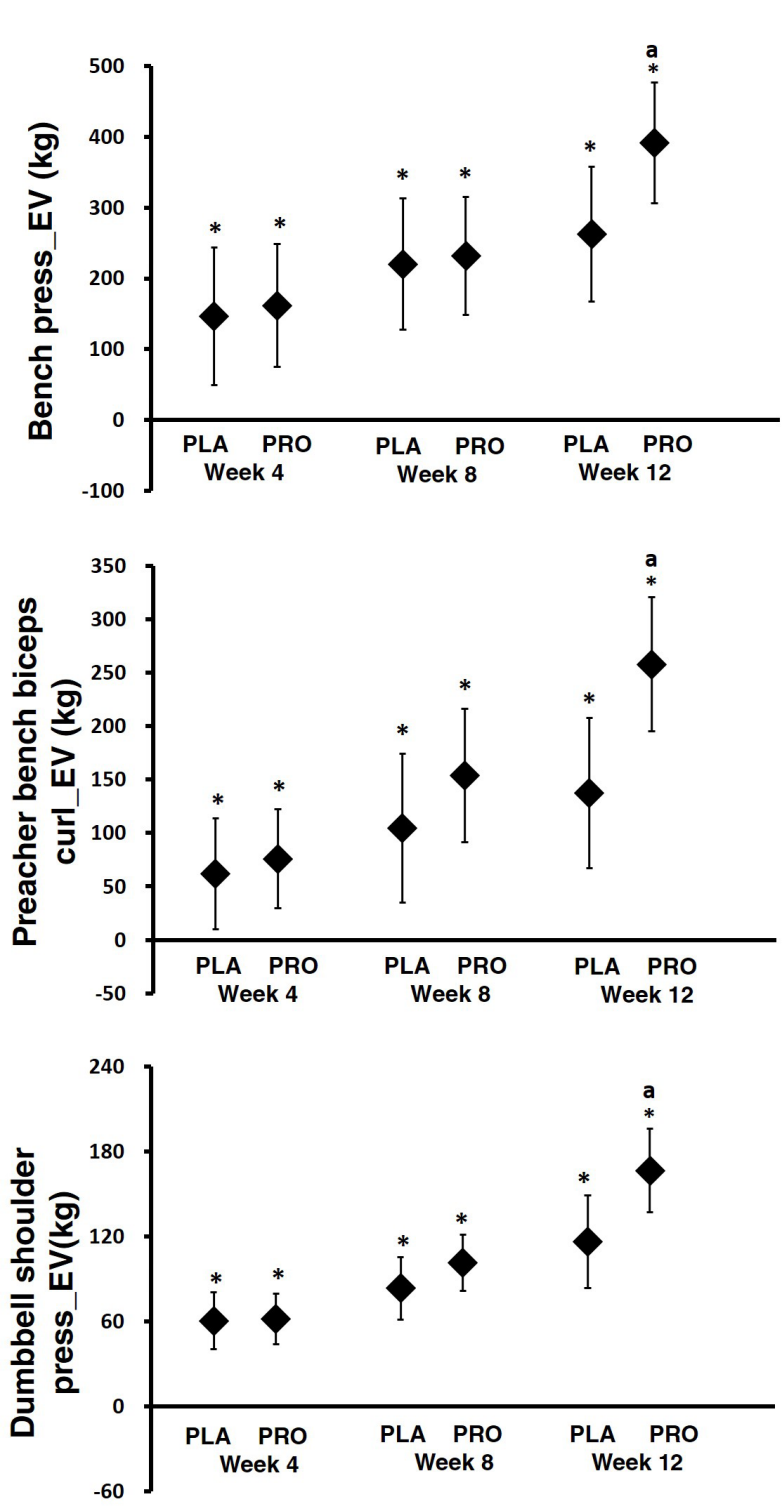
Exercise volume on upper body. Data are mean change and 95% CI. * represents *p* <0.05 difference from baseline. ^a^ represents *p* <0.05 from PLA

With regards to the exercise volume data observed during the course of the study, the upper body MANOVA analysis revealed significant time effects in changes in all variables. In addition, there were significant interactions between groups with regards to bench over barbell row (p = 0.04), preacher bench biceps curl (p = 0.01), and dumbbell shoulder press (p = 0.02). As can be seen in Figure 4, the mean changes in upper body exercise volume (EV) were generally increased, with 95% CI’s above baseline in both groups after 4, 8, and 12 weeks of intervention. For the bench press EV assessment, the 4-, 8-, and 12-week changes were as follows: PRO group (week 4: 161.50 kg, 95% CI, 74.64–248.35; week 8: 231.90 kg, 95% CI, 148.70–315.09; and week 12: 391.70 kg, 95% CI, 306.40–476.99) and PLA group (week 4: 146.65 kg, 95% CI, 49.55–243.76; week 8: 220.40 kg, 95% CI, 127.39–313.41; and week 12: 262.56 kg, 95% CI, 167.20– 357.92). The preacher bench biceps curl and dumbbell shoulder results showed a similar trend as the bench press results. Furthermore, there was a difference in bench press, preacher bench biceps curl, and dumbbell shoulder press between groups at 12 weeks.

**Figure 5.**
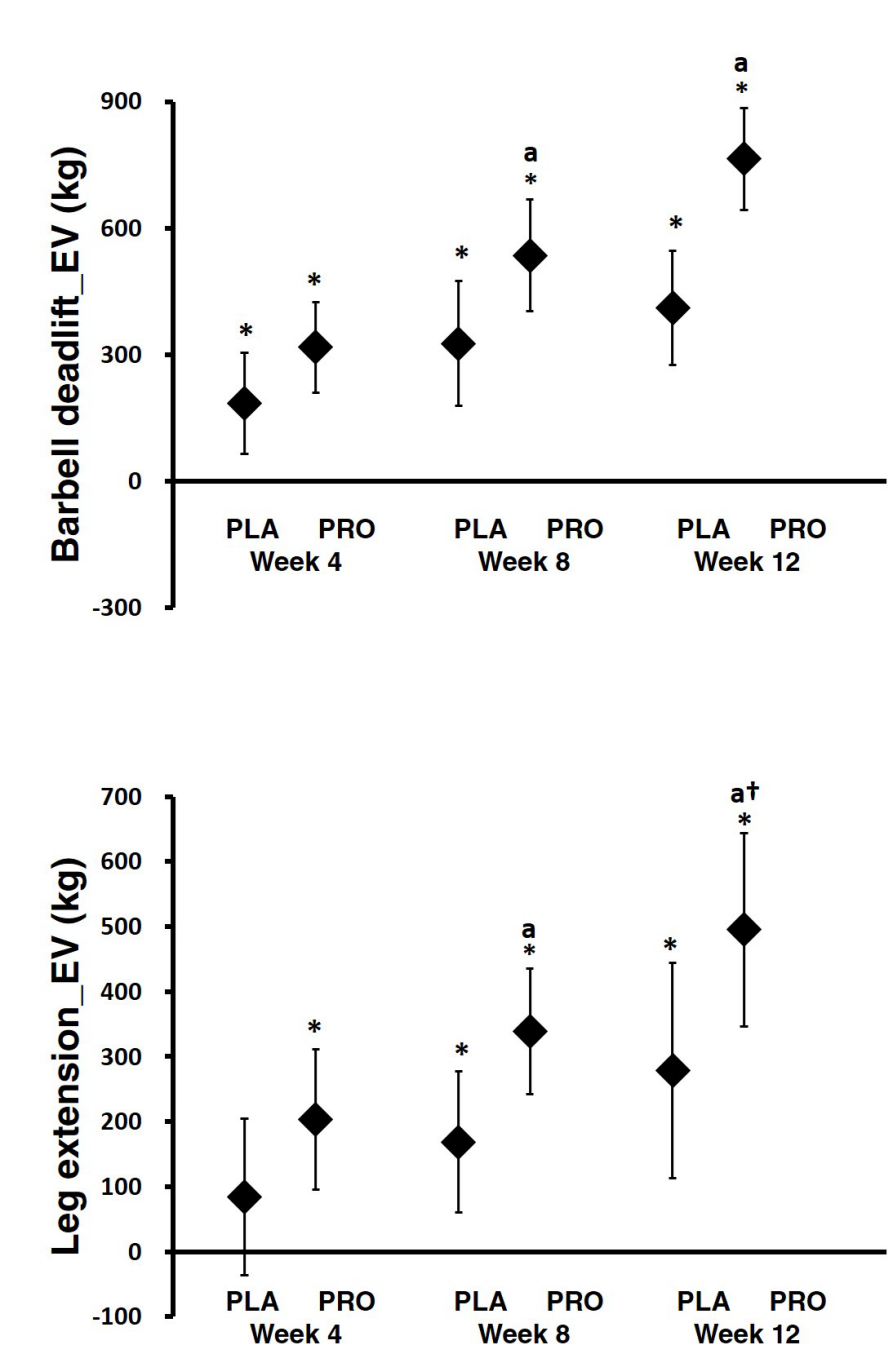
Exercise volume on lower body. Data are mean change and 95% CI. * represents* p*<0.05 difference from baseline. ^a^ represents *p*<0.05 from PLA. † represents *p*>0.05 to *p*<0.1

With regards to the lower body, MANOVA analysis revealed significant time effects in changes in all variables. There was also a significant interaction between groups in barbell deadlift (p = 0.00). As can be seen in Figure 5, the barbell deadlift EV changes at weeks 4, 8, and 12 were as follows: PRO group (week 4: 317.52 kg, 95% CI, 209.97–425.07; week 8: 535.15 kg, 95% CI, 402.69–667.60; and week 12: 764.55 kg, 95% CI, 643.50–885.59) and PLA group (week 4: 184.45 kg, 95% CI, 64.20–304.69; week 8: 326.45 kg, 95% CI, 178.36–474.54; and week 12: 411.04 kg, 95% CI, 275.71–546.38). For the leg extension EV assessment, the week 4 changes were as follows: PRO group (week 4: 203.40 kg, 95% CI, 95.89–310.90), PLA group (week 4: 84.09 kg, 95% CI, -36.09–204.28. The week 8 and 12 changes were as follows: PRO group (week 8: 339.07 kg, 95% CI, 242.30–435.84; and week 12: 495.05 kg, 95% CI, 346.49–643.60) and PLA group (week 8: 168.46 kg, 95% CI, 60.28–276.65; and week 12: 278.44 kg, 95% CI, 112.35–444.53). There was also a difference in barbell deadlift at 8 and 12 weeks and in leg extension at 8 weeks between groups. Also, there was a trend of a difference at 12 weeks in leg extension.

### Body composition 

**Figure 6.**
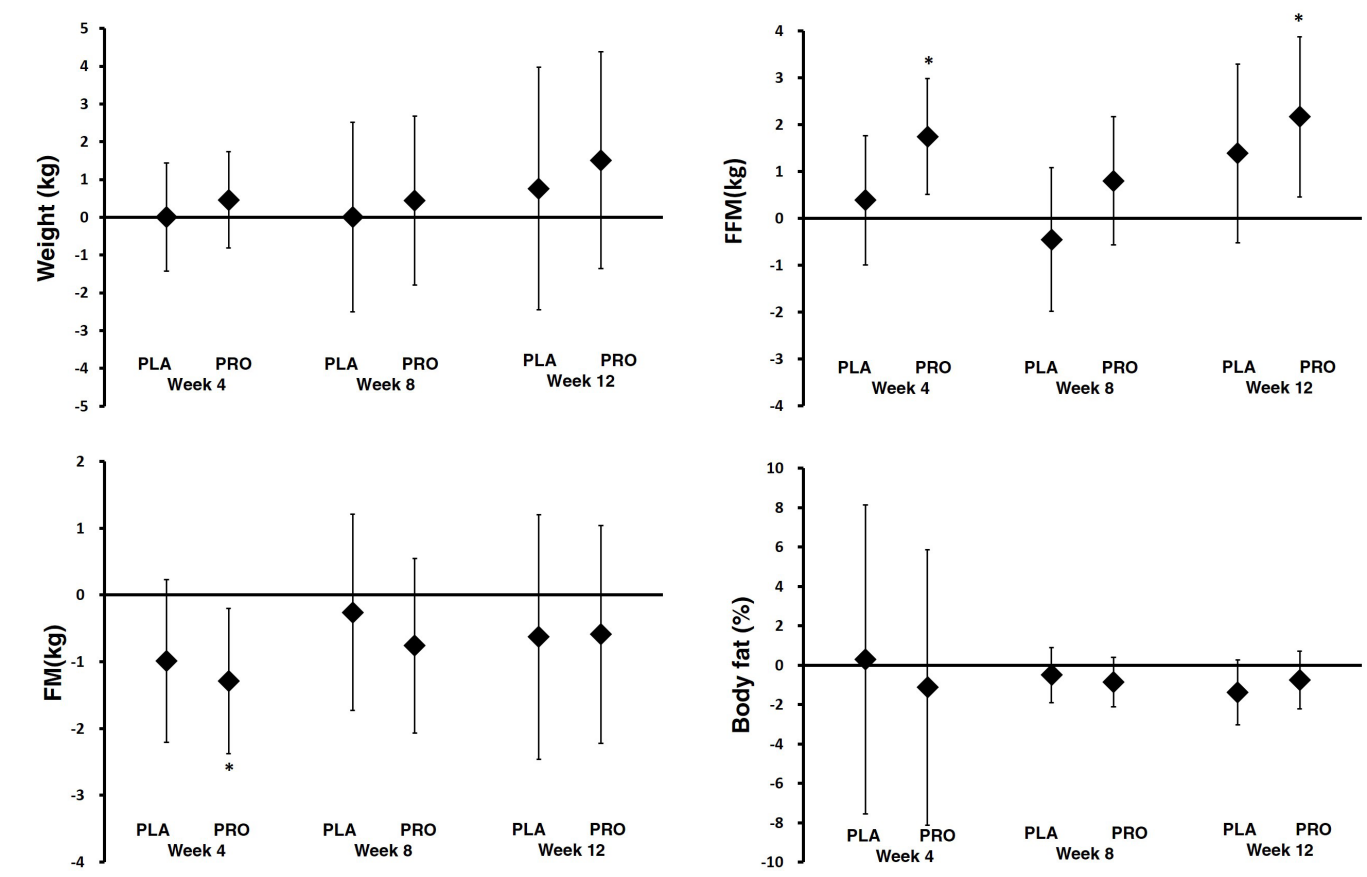
Body Composition. Data are mean change and 95% CI. * represents p <0.05 difference from baseline. ^a^ represents p <0.05 from PLA

The mean changes in body composition were generally increased, with 95% CI’s above baseline, in both groups after 4, 8, and 12 weeks of intervention (Figure 6). With regards to the FFM assessment, the week 4 and 12 changes were as follows: PRO group (week 4: 1.75 kg, 95% CI, 0.51–2.98; and week 12: 2.17 kg, 95% CI, 0.45–3.88), PLA group (week 4: 0.38 kg, 95% CI, -0.99–1.77; and week 12: 1.38 kg, 95% CI, -0.52–3.30). FFM was more increased in PRO group than PLA group at week 4 and 12. With regards to the FM assessment, the week 4 changes were as follows: PRO group (-1.29 kg, 95% CI, -2.38– -1.99), PLA group (week 4: -0.98 kg, 95% CI, -2.20–0.23). FM was more decreased in PRO group than PLA group at week 4.

### Hormone response

In terms of the hormonal response, GLM analysis revealed significant time effects in changes in IGF-1 and VEGF (Table 5). However, no significant interactions were observed between groups in terms of GH (p = 0.39), IGF-1 (p = 0.24), VEGF (p = 0.21), testo (p = 0.40), and cortisol (p = 0.85).

**Table 5.** Hormonal response

Variable	Group	Time (wk)					*p*-value
		0	4	8	12		
GH (ng/ml)	PLA	32.1 ± 2.2	32.1 ± 2.0	32.2 ± 2.1	32.0 ± 1.5	Time	0.028^†^
						Group	0.41
	PRO	30.5 ± 2.9	30.7 ± 2.4	30.9 ± 2.1	32.8 ± 2.1 ^abc^	G x T	0.010^†^
IGF-1 (ng/ml)	PLA	31.9 ± 2.1	32.0 ± 1.9	32.1 ± 1.6	32.2 ± 1.2	Time	0.008^†^
						Group	0.25
	PRO	29.8 ± 3.0	30.4 ± 2.4	30.7 ± 2.3 ^a^	32.3 ± 2.1 ^abc^	G x T	0.033^†^
VEGF (ng/ml)	PLA	52.7 ± 3.2	52.1 ± 3.6	51.8 ± 3.2	51.9 ± 1.8	Time	0.074
						Group	0.65
	PRO	51.3 ± 4.0	50.7 ± 3.9	50.9 ± 3.2	52.6 ± 3.0 ^abc^	G x T	0.037^†^
Testosterone (ng/ml)	PLA	52.6 ± 3.4	51.4 ± 3.3 ^a^	51.4 ± 2.9 ^a^	51.7 ± 2.8	Time	0.023^†^
						Group	0.75
	PRO	51.1 ± 3.8	50.8 ± 3.3	50.8 ± 2.7	52.6 ± 3.0 ^abc^	G x T	0.036^†^
Cortisol (ng/dl)	PLA	12.0 ± 2.5	12.5 ± 3.9	12.1 ± 3.3	13.1 ± 2.8	Time	0.13
						Group	0.42
	PRO	11.0 ± 1.9	11.3 ± 1.9	11.4 ± 1.8	12.9 ± 1.6	G x T	0.85

Values are presented as means ± standard deviations. All variables were analyzed by MANOVA. MANOVA analysis revealed overall Wilks’ Lambda group (p = 0.74), time (p < 0.001), and group x time (p = 0.40). Greenhouse-Geisser time and group x time interaction p-values are reported with univariate group p-values. PLA: Placebo group, PRO: Protein supplement group, GH: Growth hormone, IGF-1: Insulin growth factor-1, VEFG: Vascular endothelial growth factor * represents p < 0.05 from PLA.

^a^ represents p < 0.05 difference from baseline

^b^ represents p < 0.05 difference from wk 4

^c^ represents p < 0.05 difference from wk 8

^† ^Significant interaction or main effect, p<0.05

## DISCUSSION

The purpose of this study was to examine whether the intake of a protein blend supplement before and after resistance exercise for 12 weeks would be effective in increasing muscle function. The main finding of our study was that the PRO group had a greater improvement in muscle hypertrophy, function, circumference, strength, and endurance than the PLA group; there was also a difference in the mean change between groups at 12 weeks.

The intake of a protein supplement before and after resistance exercise sessions had a beneficial effect on muscle hypertrophy^8-9^. Lee et al.^31^ reported that the muscle circumference of the chest and thigh were significantly increased in the protein intake group, following resistance training for 12 weeks; this result is consistent with the present study. Furthermore, in the present study, the upper and lower body circumference was only improved in the PRO group, and there was a difference in the mean change from baseline in the PRO group, and between groups at 12 weeks. Moreover, although muscle strength and endurance were increased in both groups, there was a significant difference in the mean change between groups at 12 weeks, with a higher value in the PRO group than the PLA group. Thus, from these results we can deduce that the protein supplement had a positive effect on muscle hypertrophy, strength, and endurance.

These positive results were induced from dietary timing, which is very important to maximize the effect of dietary protein supplements. Hoffman and colleagues^38^ had collegiate football players who had been regularly performing resistance-training ingest 42 g of hydrolyzed protein immediately before and after exercise over the course of 10 weeks of resistance training. In this study, the timing of the protein intake did not impact on the changes in strength, power, or body composition. Moreover, Schoenfeld and colleagues^39^ examined the impact of ingesting 25 g of whey protein immediately before and after resistance training; all study participants trained 3 times a week, targeting all major muscle groups, over a 10-week period, and the authors showed that there were no differences in strength and hypertrophy between the two groups. Therefore, the ingestion of protein twice (before and after exercise) is more effective than once (before or after). These findings support that the ingestion of whey protein immediately before and immediately after workouts can promote improvements in strength and hypertrophy. Furthermore, since the subjects of Hoffman’s study^38^ were trained athletes, but the subjects of the present study were untrained, we can infer that athletes will need more protein than untrained individuals. A unique aspect of the present study was that supplementation only occurred on training days; this shows that it is an important to consume protein supplements at the optimal time in order to maximize the benefits.

The composition of the protein supplement also plays an important role in muscle hypertrophy. Shimomura et al.^13^ and Waldron et al.^32^ reported that the quantity of BCAA required to maximize muscle function, is over 5 g and the ratio of Leu:Ile:Val is 2:1:1. Leucine especially plays a critical role in muscle synthesis via mechanisms involving the mammalian target of rapamycin (mTOR) signaling pathway, which contributes to muscle protein synthesis in human skeletal muscle^33^. Furthermore, whey protein hydrolysate contains more leucine than other protein supplements, and results in increased muscle hypertrophy and function^6^. In the present study, the composition of the test solutions was as follows: a BCAA solution containing 5.8 g of BCAA mixture (Leucine:Isoleucine:Valine = 2:1:1) and whey protein hydrolysate. This composition resulted in muscle hypertrophy and improvement of muscular function.

In terms of the food intake results, the PRO group maintained a comparable level of protein uptake by 8 weeks compared to the PLA group, with a steadily increasing protein intake. However, there was no improvement in muscle hypertrophy and muscular function in proportion to the usual protein intake in the PLA group. Additionally, the total protein intake of the PRO group was similar to the Dietary Reference Intakes for Koreans (KDRIs), which is 65 g per day for men aged 19–29 years^40^. Moreover, the energy intake including the quantity of protein supplement was not only increased in the PRO group, but it was also less than that in the PLA group. Thus, protein supplementation was necessary to improve muscle function. Furthermore, the protein supplements were only provided on training days, suggesting that the timing and composition of protein intake are more important than the total amount.

Based on the general theory, muscle strength is increased rapidly in the early stages of the resistance training period as a result of neurotic factors, and then is further increased with the improvement of muscle hypertrophy at a later stage (i.e. after 6 weeks)^37^. In this study, muscle strength increased steadily in both groups until the later part of the RET. The significant difference between groups appeared at 12 weeks in some of the upper and lower body variables. Also, muscle circumference showed a difference in mean change between groups at 12 weeks, and the value was higher in the PRO group than the PLA group. Thus, the increase in muscle strength in the later stage results from the enhanced muscle circumference. It is generally considered that protein supplementation has a positive effect on muscle function and should be consumed for more than 12 weeks during resistance exercise. However, the present study showed that although muscle hypertrophy was increased, there was no difference in FFM between the groups. In addition, muscle circumference was measured with a morphological estimation method. In human studies, muscle hypertrophy resulting from an increase in muscle fiber size is determined by measuring the cross-sectional area^37^. Therefore, it is necessary to measure the cross-sectional muscle area using MRI and CT to verify the increasing muscle fiber size and to assess the molecular biological methods, such as mTOR signaling, through a muscle biopsy. 

The change in muscle function results from the hormonal response (i.e. GH, IGF-1, VEGF, testo, and cortisol), neuromuscular adaptation, and muscle hypertrophy. In the present study, there was no interaction effect on the hormonal response. This can be explained from two perspectives. Firstly, previous studies have shown that protein supplementation combined with RET significantly increased the anabolic hormonal response, such as GH, IGF-1, VEGF, and testo, which resulted in an improvement in muscle function^34^. Kraemer et al.^5^ reported that resistance exercise with PRO-CHO blend supplementation before and immediately after exercise increased the anabolic hormone at concentration at 0 min post-exercise, which was decreased by 60 min post-exercise. In our study, we observed no significant interaction effect in all blood parameters, most likely because we measured the resting values 48 hours post-exercise. Based on a previous study, it seems that the concentration of anabolic hormone was decreased on the test day (48 hours post-exercise)^36^. Secondly, the resting concentration of GH and IGF-1 was not increased in untrained young men after 10 weeks resistance training^31^. With regards to the static anabolic hormonal response, this may be due to the increased sensitivity of the receptor to the membrane of the skeletal muscle.

## CONCLUSION

The protein intake through regular meals was increased steadily during exercise training in the PLA group. However, there was no improvement in muscle hypertrophy and muscular function in proportion to the protein intake. In addition, most of the previous studies provided protein supplement over five times per week, whereas the present study only provided on training days. Thus, protein supplementation is essential to the improvement of muscule function in response to RET. Furthermore, the composition and timing of protein intake are more important than the total amount.
